# Effect of Precipitation Change on Desert Steppe Aboveground Productivity

**DOI:** 10.3390/biology14081010

**Published:** 2025-08-06

**Authors:** Yonghong Luo, Jiming Cheng, Ziyu Cao, Haixiang Zhang, Pengcuo Danba, Jiazhi Wang, Ying Wang, Rong Zhang, Chao Zhang, Yingqun Feng, Shuhua Wei

**Affiliations:** 1 School of Biological Science and Engineering, North Minzu University, Yinchuan 750021, China; 21915013@mail.imu.edu.cn (Y.L.); chengjiming@mails.ccnu.edu.cn (J.C.); czy200218@163.com (Z.C.); 19113848044@163.com (P.D.); 2 Ningxia Academy of Agriculture and Forestry Sciences, Plant Protection Institute, Yinchuan 750002, China; wangying108@163.com (Y.W.); yczhrnx@163.com (R.Z.); 3 College of Grassland Science and Technology, China Agricultural University, Beijing 100193, China; haixiangzhang@cau.edu.cn; 4 Chengde Meteorological Disaster Prevention Center of Hebei Province, Chengde 067000, China; george0602@126.com; 5 School of Life Sciences, Central China Normal University, Wuhan 430079, China; zhangchao2023@mails.ccnu.edu.cn (C.Z.); fyq0016094@163.com (Y.F.)

**Keywords:** biodiversity, desert steppe, functional traits, precipitation change, aboveground productivity

## Abstract

Precipitation changes have significant impacts on biodiversity and ecosystem productivity. However, the effects of precipitation changes on species diversity have been the focus of most previous studies. Little is known about the contributions of different dimensions of biodiversity (species, functional, and phylogenetic diversity) in linking long-term precipitation changes to ecosystem functions. In this study, a randomized design was conducted in the desert steppes of Ningxia, which included three treatments: natural rainfall, precipitation reduced by 50%, and precipitation increased by 50%. After 4 years of treatment, the effects of precipitation changes on aboveground productivity and its underlying mechanisms were explored. The results showed that (1) reduced precipitation significantly decreased phylogenetic diversity and species diversity, but had no significant effect on functional diversity; (2) reduced precipitation significantly decreased aboveground productivity, while increased precipitation significantly enhanced aboveground productivity; and (3) changes in precipitation primarily regulated aboveground productivity by altering soil nitrogen availability and the size of dominant plant species. This study provides important theoretical and practical guidance for the protection and management of desert steppe vegetation under future climate change.

## Simple Summary

Precipitation changes have a significant impact on grassland productivity. However, little is known about how different dimensions of biodiversity connect to the impact of precipitation changes on grassland productivity. A controlled experiment was conducted to explore the impact of precipitation changes on the aboveground productivity of desert steppe. It was found that a ±50% change in precipitation significantly altered the aboveground productivity of the plant community. More importantly, we discovered that precipitation changes mainly regulate the aboveground productivity of desert steppe plants by altering soil moisture and inorganic nitrogen, as well as adjusting the plant height of dominant species.

## 1. Introduction

Against the backdrop of global climate change, the precipitation pattern is undergoing remarkable and complex changes [1,2]. These changes have exerted a substantial impact on the functions of desert steppe ecosystems and have attracted extensive attention from ecologists [3,4,5]. Desert steppes, as a typical ecosystem type in arid and semi-arid regions, exhibit high sensitivity to precipitation changes due to their unique geographical location and fragile habitat conditions [4]. In recent years, studies have confirmed that fluctuations in precipitation, both increases and decreases, not only directly affect the availability of soil moisture but also indirectly influence the material cycle and energy flow of desert grassland ecosystems by altering soil nutrients and plant community composition [5,6,7]. Moreover, as a crucial ecological barrier connecting arid regions and semi-humid regions, the stability of the functions of desert steppe ecosystems is directly related to the maintenance of regional biodiversity, the improvement of carbon sequestration capacity, and the guarantee of ecological security [8,9]. Therefore, systematically exploring the impact mechanism of precipitation changes on the functions of desert steppe ecosystems plays an important role in revealing the response patterns of ecosystems in arid and semi-arid regions under the background of global change.

The impact of precipitation changes on community productivity has received extensive attention in grassland ecosystems [9,10,11]. Generally, drought conditions reduce grassland productivity [12,13,14]. This is because drought decreases soil moisture, inhibits the synthesis of plant organic matter [15,16], reduces soil enzyme activity and the available nutrients [17], and restricts plants’ acquisition of nutrients [17]. However, there are also some different research findings regarding the impact of drought on productivity. For example, in the alpine meadows of Switzerland, three consecutive years of drought did not cause a significant change in productivity [18]. Even the results of a study on an upland grassland ecosystem showed that drought can increase the productivity of plant communities [19]. There are relatively few studies investigating increased precipitation (or irrigation) [20,21]. A previous global meta-analysis showed that ecological experiments with only supplemental watering did not alter productivity [22]. Meanwhile, results from a field control experiment demonstrated that increasing water supply can significantly enhance plant community productivity [23]. However, most current controlled field experiments have only explored the impacts of growing-season precipitation changes on productivity, while the effects of year-round manipulated precipitation on grassland productivity remain understudied. This limitation restricts our understanding of ecosystem responses to climate change across temporal scales.

Biodiversity, serving as a critical mechanism regulating community productivity responses to global change, has seen increasing focus on the mechanistic roles of its multidimensional attributes [14,24,25]. First, species richness exerts significant positive effects on productivity through niche differentiation and enhanced resource use efficiency, a pattern corroborated by extensive experimental data [26,27,28]. Second, phylogenetic diversity, which integrates functional information embedded in species evolutionary histories, has been validated as an effective predictor of plant productivity [29,30]. Notably, functional diversity, through reflecting interspecific complementarity and differential resource use efficiency, emerges as a key driver explaining community productivity dynamics [29]. Moreover, accumulating research findings indicate that community-weighted mean traits serve as a robust explanatory framework for ecosystem functioning [14,31]. This metric, frequently employed to quantify plant functional diversity, principally captures the effects of dominant species on ecosystem processes [32]. Although existing research has individually illuminated the roles of different biodiversity dimensions, there remains a lack of integrative analysis combining species richness, phylogenetic diversity, and functional diversity metrics, creating significant research gaps in mechanistically understanding ecosystem responses to precipitation changes.

Desert steppe, as the main type of grassland ecosystem in arid and semi-arid regions, plays a vital role in maintaining biodiversity and preventing land degradation and desertification [33]. It also provides forage resources for local communities, supporting the development of the livestock industry, and serves as an important safeguard for ecological balance and human well-being [34]. This type of grassland has long been constrained by water availability [35], and climate models predict that the frequency of precipitation changes in this region will also increase [36]. This study seeks to (1) examine the impact of ±50% changes in precipitation on plant diversity and aboveground productivity, and (2) determine biotic and abiotic mechanisms that cause these changes.

## 2. Materials and Methods

### 2.1. Overview of the Research Site

**Figure 1:**
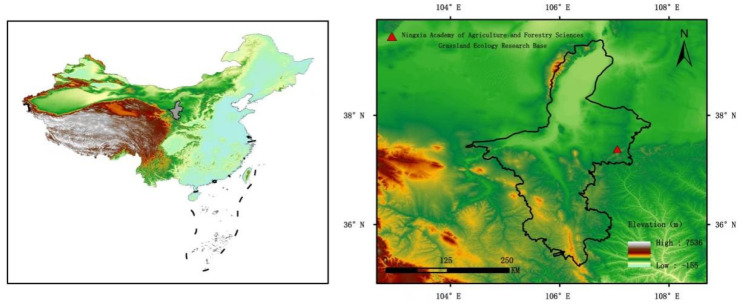
Location of the experimental research site (China’s Standard Administrative Division Data GS (2024) No. 0650).

This study was conducted at the Grassland Ecology Research Base of the Institute of Plant Protection, Ningxia Academy of Agricultural Sciences, in Yanchi County, Ningxia Hui Autonomous Region (106°95′ E, 37°43′ N, altitude 1146 m) (Figure 1). Yanchi County is connected to the Mu Us Desert in the north and borders the Loess Plateau in the south. It is a transitional zone from typical steppe to desert steppe and has a typical temperate continental climate.

In the research area, there is a large temperature difference between day and night. The winter is cold and long, with the average temperature in January usually below −10 °C. The summer is relatively hot but short, and the average temperature in July is generally around 22 °C. The temperature changes rapidly in spring and autumn, with obvious warming or cooling. The average annual rainfall is 289 mm, which is concentrated from July to September, accounting for about 60–70% of the annual precipitation.

The zonal soil in the research area is mainly light gray cinnamon soil, with a sandy loam and silty loam texture, and the pH is around 8.8. The vegetation in this area mainly consists of mesophytic and xerophytic perennial herbaceous plants. *Lespeseza potaninii*, *Agropyron mongolicum*, *Polygala tenuifolia*, *Artemisia gansuensis*, *Artemisia scoparia*, etc., are the main dominant species.

### 2.2. Experimental Design

**Figure 2:**
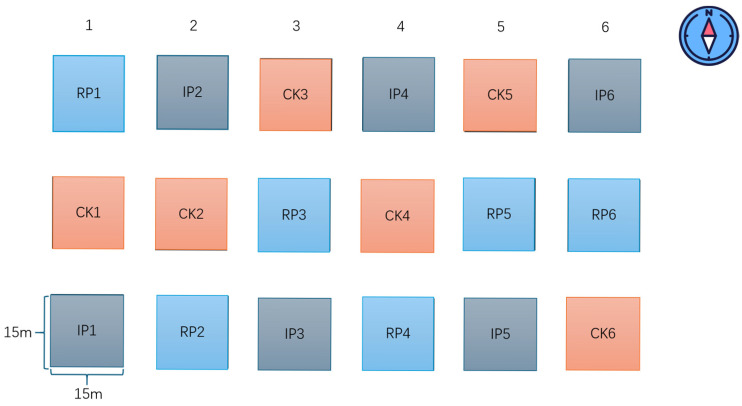
Plan layout diagram of experimental plots for precipitation change experiments. Note: CK, RP, and IP represent natural precipitation, 50% reduced precipitation, and 50% increased precipitation, respectively.

This study was conducted on the “Precipitation Change” treatment platform within the Grassland Ecology Research Base of the Institute of Plant Protection, Ningxia Academy of Agriculture and Forestry Sciences. The platform was established in 2021. Three precipitation treatments were set up using a random assignment method, including control receiving natural precipitation (CK), 50% reduced precipitation (RP), and 50% increased precipitation (IP). Each plot had an area of 15 m × 15 m, and there was a 2 m buffer zone between adjacent plots. In the plots where the rainfall is reduced by 50%, a rain shelter of 15 m × 15 m was installed (with a roof height of 2 m and an edge height of 1.2 m). The roof of the rain shelter is made of transparent polyethylene strips with a width of 20 cm and a density of 1/2 (with a light transmittance of over 90% and no significant impact on soil temperature [14]). Both sides of the transparent polyethylene strips are folded up by approximately 1 cm to prevent the intercepted precipitation from flowing away from the sides. The transparent polyethylene strips were fixed to the iron frame with screws in a V-shape, and the lower end of the transparent polyethylene was connected to a water trough, which intercepts about 50% of the rainfall in each rainfall event. Fifty percent increased precipitation treatment: After each precipitation event, the rainwater intercepted in each plot with 50% precipitation reduction was evenly sprayed onto the corresponding adjacent plot with 50% precipitation increase via an automatic sprinkler device. Each treatment had 6 replicates, resulting in a total of 18 treatment plots (Figure 2). The annual precipitation from 2021 to 2024 was 227.9 mm, 313.2 mm, 264.1 mm, and 332.4 mm; 50% reduced precipitation was 113.95 mm, 156.6 mm, 132.05 mm, and 166.2 mm; and 50% increased precipitation was 341.85 mm, 469.8 mm, 396.15 mm, and 498.6 mm (Table 1).

#### 2.2.1. Investigation of Plant Communities and Determination of Functional Traits

In the middle of July 2024, in each of the 18 plots (3 treatment methods × 6 replicates), a fixed 1 m × 1 m quadrat was randomly set up. These fixed quadrats were placed away from the edges of the plots and used for conducting plant community surveys. The number of plant species (richness) in each permanent quadrat was recorded. In late July, plant height (PH), leaf area (LA), specific leaf area (SLA), leaf dry matter content (LDMC), stem dry matter content (SDMC), and stem leaf ratio (SLR) of each species was examined within a 1 m × 1 m iron frame randomly placed in each plot. In late August, a 1 m × 1 m quadrat was randomly selected in each treatment plot. After removing litter, the aboveground tissues of plants within the quadrat were cut off at ground level by species, placed in envelopes, and dried in an oven at 65 °C to a constant weight. Their mass was measured using a one-thousandth balance, and the sum of the dry weights of all species in the plot was taken as the aboveground net primary productivity [14].

Plant height of each species was measured as the average height of five randomly selected individuals, and all individuals were measured for species with fewer than five individuals. The aboveground plant parts within the iron frame were collected by species to measure LA using a scanner (with Sigmascan 4.1), fresh leaf weight, and stem weight. The plant tissues were then dried to constant weight for calculating SLA, LDMC, SDMC, and SLR. Specific leaf area (SLA) = leaf area (LA)/leaf dry weight (LD); leaf dry matter content (LDMC) = LD/fresh leaf weight (LF); stem dry matter content (SDMC) = stem dry weight (SD)/fresh stem weight (SF); and stem leaf ratio (SLR) = stem dry weight (SD)/leaf dry weight (LD). Community-weighted means (CWM) of plant traits and functional dispersion (FDis) were used to quantify plant functional diversity. Community-weighted means (CWM) of plant traits were calculated as follows (Lavorel et al. [37]):

(1) $$CWM=\sum P_{i}\times {trait}_{i}$$

where *P_i_* is the relative aboveground biomass of species *i* in the community, and *trait_i_* is the trait value of species *i*. The CWM is closely related to the “mass ratio hypothesis” [38], which proposes that ecosystem functions or processes are largely determined by the dominant species’ functional traits. We calculated functional dispersion (*FD_is_*) for six traits together, following Laliberte and Legendre [39].

(2) $${FD}_{is}=\sum b_{j}z_{j}/\sum b_{j}$$

where *b_j_* is the biomass of species *j*, and *z_j_* is the distance of species *j* to the weighted centroid c, calculated as:

(3) $$c=\sum b_{j}x_{ij}/\sum b_{j}$$

where *x_ij_* is the attribute of species *j* for trait *i*.

To measure phylogenetic relationships between plant species in the experimental area, we used maximum likelihood to construct a phylogenetic tree that constrains family-level phylogenies to the APG III classification system [40]. Community phylogenetic diversity was quantified using the phylogenetic diversity (PD). PD represents the total length of evolutionary branches of species in a community on a phylogenetic tree, calculated as:

(4) $$PD=B\times \frac{\sum_{i}^{B}L_{i}A_{i}}{\sum_{i}^{B}A_{i}}$$

where *B* is the number of branches in the tree, $L_{i}$ is the length of branch i, and $A_{i}$ is the relative aboveground biomass of species sharing branch i [41].

#### 2.2.2. Determination of Soil Moisture and Inorganic Nitrogen

During each growing season (from May to September), soil sampling was carried out at regular intervals. Specifically, every two weeks, three soil cores, each with a diameter of 2 cm and a depth of 10 cm, were obtained from each plot beyond the permanent quadrat. After sampling, the holes were immediately filled with soil sourced from outside the experimental area. To determine the soil moisture content, the collected soil cores were first weighed and then placed in an environment at 105 °C for 36 h to dry before being weighed again. In addition, in the latter parts of July and August annually, five soil cores, each with a diameter of 2 cm and a depth of 10 cm, were randomly selected from each plot. These cores were then combined to form a single composite sample. After fresh soil samples were extracted with 1 mol/L potassium chloride (KCl) solution, the concentrations of ammonium nitrogen and nitrate nitrogen in the soil were accurately determined using a flow injection analyzer (manufactured by FossTecator, Hillerød, Denmark), and then the concentration of inorganic nitrogen in the soil was calculated.

Soil moisture content = (fresh soil weight − dry soil weight)/dry soil weight × 100%(5)

Soil inorganic nitrogen = soil ammonium nitrogen + soil nitrate nitrogen(6)

### 2.3. Statistical Analysis of Data

We first used linear mixed-effects models (LMMs) to analyze the effects of precipitation changes (CK, RP, and IP) on aboveground net primary productivity, plant diversity, and soil properties. In this model, aboveground net primary productivity (ANPP), plant biodiversity, and soil properties were set as the fixed effects, and plot was treated as the random effect to control the non-independence among plots. The model analysis was implemented in R version 3.6.3 using the lmer() function from the lme4 package. After model validation, we further conducted pairwise comparisons of differences among the three treatments using Tukey’s HSD post hoc multiple comparison test (via the emmeans package). Significant differences (*p* < 0.05) were marked with different uppercase letters in the figure. Then, a general linear regression model was applied to analyze the relationships between plant biodiversity, soil properties, and ANPP. Finally, a random forest model was utilized to assess the explanatory power of biotic and abiotic factors for ANPP. The random forest model was implemented in R version 3.6.3 using the randomForest package, with the number of regression trees set to 500 (ntree = 500), and the number of variables randomly sampled as candidates at each split (mtry) automatically selected using the default method. Model stability was evaluated using 10-fold cross-validation. Variable importance was ranked based on the “percent increase in mean squared error” (%IncMSE), and significance (*p*-values) was assessed via permutation tests.

## 3. Result

### 3.1. Effects of Alterations in Precipitation on Aboveground Net Primary Productivity

**Figure 3:**
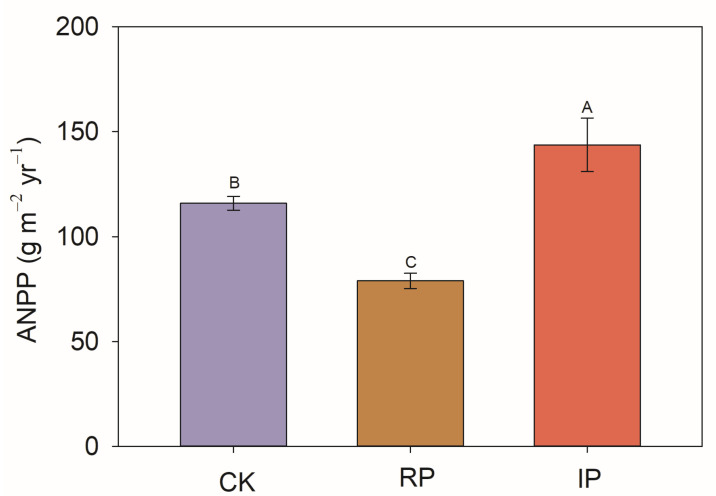
The impact of precipitation changes on aboveground net primary productivity (ANPP). Note: Different uppercase letters indicate significant differences (*p* < 0.05). CK, RP, and IP represent natural precipitation, 50% reduced precipitation, and 50% increased precipitation, respectively.

Reducing precipitation by half significantly decreased aboveground net primary productivity (a decrease of 32%). Conversely, increasing precipitation by half significantly enhanced aboveground net primary productivity (an increase of 24%) (Figure 3).

### 3.2. The Responses of Soil Water and Soil Inorganic Nitrogen to Precipitation Changes

**Figure 4:**
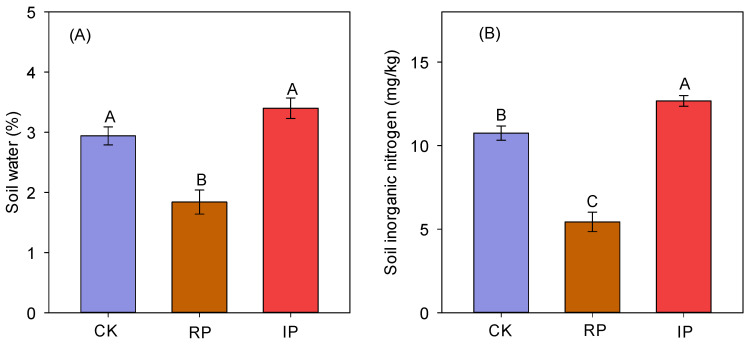
Responses of soil moisture and inorganic nitrogen to precipitation changes. Note: (**A**) soil water (%); (**B**) soil inorganic nitrogen (mg/kg); Different uppercase letters indicate significant differences (*p* < 0.05). CK, RP, and IP represent natural precipitation, 50% reduced precipitation, and 50% increased precipitation, respectively.

Reducing precipitation by half significantly decreased soil moisture and soil inorganic nitrogen (Figure 4A,B). However, increasing precipitation by half had no significant impact on soil moisture but significantly increased soil inorganic nitrogen (Figure 4A,B).

### 3.3. The Impact of Precipitation Changes on Biodiversity

**Figure 5:**
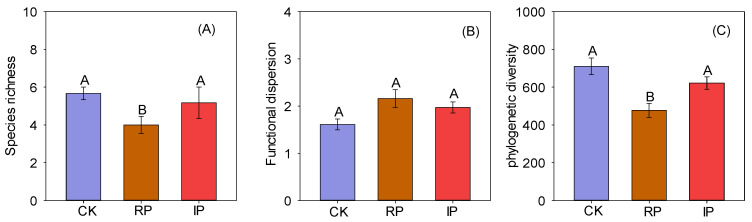
Responses of plant diversity to precipitation changes. Note: Different uppercase letters indicate significant differences (*p* < 0.05). CK, RP, and IP represent natural precipitation, 50% reduced precipitation, and 50% increased precipitation, respectively.

**Figure 6:**
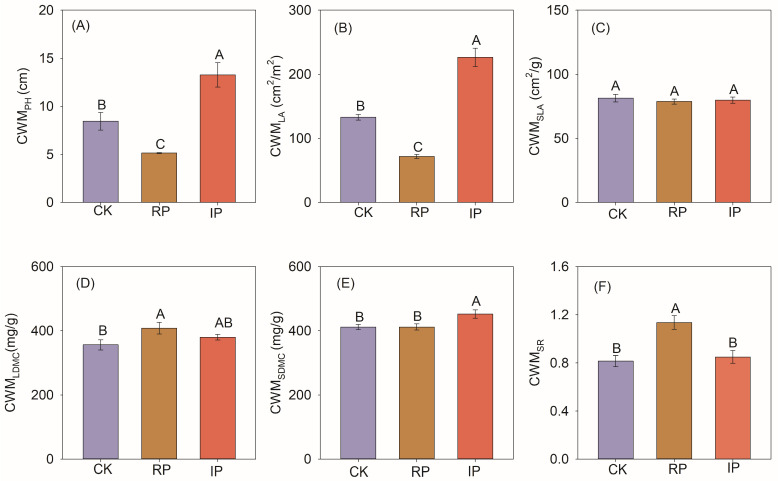
Responses of functional traits of plant communities to precipitation changes. Note: Different uppercase letters indicate significant differences (*p* < 0.05). CK, RP, and IP represent natural precipitation, 50% reduced precipitation, and 50% increased precipitation, respectively. (**A**–**F**) represent the community-weighted mean values of plant height (CWM_PH_), leaf area (CWM_LA_), specific leaf area (CWM_SLA_), leaf dry matter content (CWM_LDMC_), stem dry matter content (CWM_SDMC_), and stem-to-leaf ratio (CWM_SLR_), respectively.

Reducing precipitation by half significantly decreased species richness and phylogenetic diversity (Figure 5A,C), but had no significant impact on functional dispersion (Figure 5B). Reducing precipitation by half significantly reduced the community-weighted mean values of plant height (CWM_PH_) and leaf area (CWM_LA_) (Figure 6A,B), and significantly increased the community-weighted mean values of leaf dry matter content (CWM_LDMC_) and stem-leaf ratio (CWM_SR_) (Figure 6D,F). Increasing precipitation by half significantly increased the CWM_PH_, CWM_LA_, and CWM_SDMC_ (Figure 6A,B,E).

### 3.4. The Associations Among Plant Biodiversity, Soil Properties, and Aboveground Net Primary Productivity

**Figure 7:**
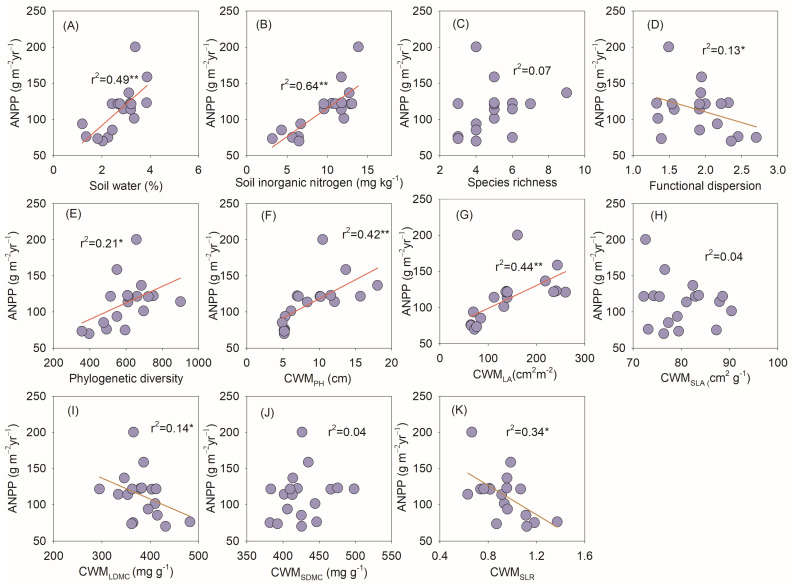
The relationships among plant biodiversity, soil properties, and aboveground net primary productivity (ANPP). Note: (**A**–**E**) represent the relationships among soil water, soil inorganic nitrogen, species richness, functional dispersal, phylogenetic and ANPP (**F**–**K**) represent the relationships among the community-weighted mean values of plant height (CWM_PH_), leaf area (CWM_LA_), specific leaf area (CWM_SLA_), leaf dry matter content (CWM_LDMC_), stem dry matter content (CWM_SDMC_), stem-to-leaf ratio (CWM_SR_) and ANPP. * *p* < 0.05 & ** *p* < 0.01.

Soil moisture, soil inorganic nitrogen, phylogenetic diversity, CWM_PH_, and CWM_LA_ were positively correlated with aboveground net primary productivity (ANPP) (Figure 7A,B,E–G), while functional dispersion, CWM_LDMC_, and CWM_SR_ were negatively correlated with aboveground net primary productivity (ANPP) (Figure 7D,I,K).

### 3.5. The Explanatory Ability of Biodiversity and Soil Properties with Respect to Aboveground Net Primary Productivity

**Figure 8:**
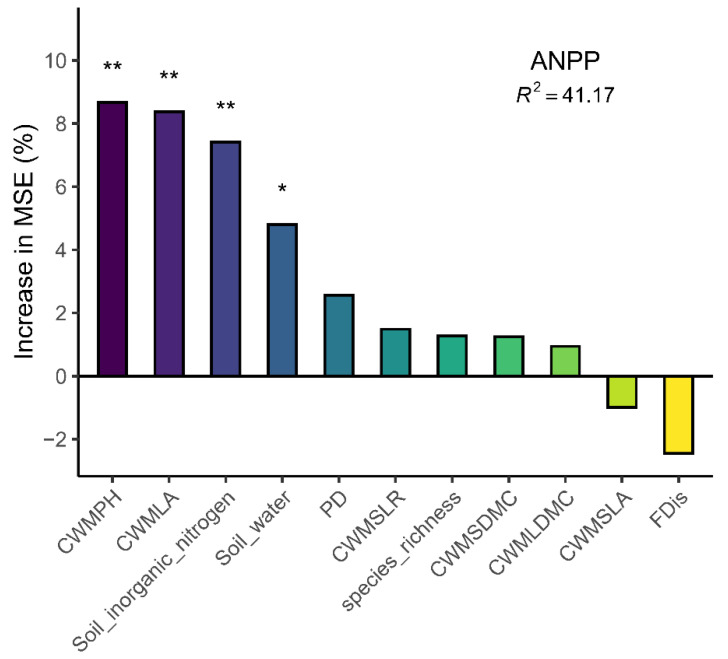
Explanation of the aboveground net primary productivity (ANPP) by biotic factors and abiotic factors. Mean Squared Error (MSE), *, ** indicates that the interpretation of the independent variable has a significant effect on the dependent variable (* *p* < 0.05, ** *p* < 0.01).

The model explains 41.17% of the variation (R^2^ = 41.17). Among the variables, those contributing the most to the model error (MSE) are, in order, community-weighted mean plant height (CWM_PH_), leaf area (CWM_LA_), soil inorganic nitrogen content, and soil water content, all of which have a significant positive impact on ANPP (* *p* < 0.05, ** *p* < 0.01). (Figure 8).

## 4. Discussion

After four years of controlled precipitation treatments in desert steppes, it was found that a 50% reduction decreased the aboveground productivity of the plant community by 32%. Conversely, a 50% increase in precipitation enhanced the aboveground productivity of the plant community by 24%. An increase in precipitation of 50% had no significant effect on species richness, while a 50% reduction in precipitation significantly decreased species richness. By analyzing soil properties and plant diversity, the study explored the mechanisms underlying the effects of altered precipitation on the aboveground productivity of plant communities. It was found that precipitation changes primarily drove aboveground productivity by altering plant functional traits (plant height and leaf area), as well as soil inorganic nitrogen content and soil moisture.

### 4.1. Effects of Precipitation Changes on Aboveground Productivity

Many previous studies have confirmed that drought can significantly reduce the productivity of grassland ecosystems [14,42,43]. Our research results are consistent with the above reports. Reducing precipitation by 50% significantly decreases the aboveground productivity of desert grasslands (decreased by 32%) (Figure 3). This is because plants in the study area are strongly limited by water availability. Under drought conditions, soil water content decreases, leading to insufficient water available for plants [44]. This affects normal physiological processes in plants such as photosynthesis and transpiration, hinders their growth and development, and thereby reduces productivity [14]. In addition, drought can also affect the dissolution and transfer of soil nutrients, limiting plant roots from absorbing sufficient nutrients [45]. Finally, drought reduces the net carbon assimilation rate of leaves, limits cell division and elongation, and increases tissue mortality [46]. Increasing precipitation by 50% improves aboveground productivity (increased by 24%) (Figure 3). The reason lies in that increased precipitation raises soil water content and promotes the accumulation of soil nutrient content [1], being beneficial to plant growth and development. In addition, our research results reveal that the negative impact of drought years on the productivity of desert steppes is extremely severe. We call on relevant authorities to increase artificial precipitation when favorable precipitation opportunities arise, thereby mitigating the negative effects of drought on productivity and maintaining and protecting the relevant ecological functions of the desert steppe.

### 4.2. Effects of Precipitation Changes on Biodiversity

Although dozens of studies on the effects of manipulated precipitation changes on grassland plant biodiversity have been conducted [12]. Globally, the relevant results vary, which is due to differences in grassland ecosystems across different regions [43]. Most experimental evidence suggests that the response of biodiversity varies with the magnitude and direction of manipulated precipitation [12,47,48], and depends on whether the experiment involves increased or decreased water [48]. The results of this study showed that reducing precipitation by 50% significantly decreased the species richness of plant communities (Figure 5A). Desert steppe plants are highly dependent on water. Reduced precipitation directly induces soil drought, disrupts plant water homeostasis, and constrains seed germination of annual and biennial herbaceous species dependent on shallow water resources [49]. Additionally, under drought conditions, limited water resources trigger more intense competition among species. Dominant species, particularly those with strong drought tolerance, occupy more resources (such as water, light, and nutrients), thereby squeezing the ecological niches of other species and resulting in the exclusion of vulnerable [50], and decreasing plant-available nutrients, limiting growth. Functional diversity, which reflects the complementary effects among species, is regulated by precipitation changes [29]. This study found that precipitation changes had no significant effect on functional diversity (Figure 5B). This may be related to the limited number of species, scarce water resources, and low interspecific differentiation in our study area [14]. Phylogenetic diversity reflects the diversification history of species in the evolutionary tree, and studies have shown that phylogenetic diversity is also affected by precipitation changes [51]. Our results showed that a 50% reduction in precipitation significantly decreased phylogenetic diversity (Figure 5C). This may be related to the fact that drought has reduced the relative abundance of forbs and grasses (Supplementary Table S1).

### 4.3. Mechanisms of Productivity Impacts by Precipitation Changes

Species richness plays an important role in regulating the productivity of plant communities. Many existing experimental evidences show that species richness has a positive effect on productivity [50,52,53]. However, our research results showed that species richness had no effect on the aboveground productivity of plant communities (Figure 7C). This was because there were few species in the study area, and precipitation changes had little impact on species’ richness. This is consistent with the results of previous studies [14,54]. Phylogenetic diversity also plays an important role in predicting changes in community productivity [55]. Although our study confirmed in general linear regression analysis that phylogenetic diversity was weakly negatively correlated with community productivity, random forest models showed phylogenetic diversity poorly predicted community productivity changes (Figure 7E). Similarly, functional dispersion had a low explanatory power for community productivity (Figure 7D). In the desert steppe ecosystem, plants were subjected to severe water limitation, which induced a convergence of ecological niches and weak interspecific complementarity in resource utilization [32]. As a result, functional diversity exhibited limited capacity to predict variations in productivity.

Plant height and leaf area represent critical functional traits of plant species, embodying their ecological strategies in contrasting habitats, including light interception, carbon assimilation, and competitive competence [56]. Our research findings demonstrate that the community-weighted mean (CWM) of plant height and leaf area serves as a robust explanatory factor for variations in aboveground productivity across plant communities. Our results further support the biomass ratio hypothesis, which states that the traits of dominant species play a critical role in regulating ecosystem functions, while ecosystem functions are insensitive to species richness [32]. In arid and semi-arid regions, water is the most critical ecological factor limiting normal photosynthesis and inhibiting plant cell growth. Increases or decreases in water availability have opposite effects on plant growth [53]. Moreover, precipitation changes can alter soil microbial activity, impact the availability of soil organic matter and available nitrogen, and modify plant nutrient uptake [24]. However, previous biodiversity and ecosystem functioning(BEF) experiments indicated that it is species richness that affects community productivity due to the existence of compensation among species [57,58,59]. This differs from the findings of our study, which can be attributed to the fact that the study area has fewer species with uneven distribution, and thus, ecosystem functions are mainly regulated by dominant species.

## 5. Conclusions

Through four years of controlled treatments (50% increased precipitation and 50% reduced precipitation) in desert steppes, consistent with previous studies, we found that the two treatments had opposite effects on the aboveground productivity of plant communities. Additionally, 50% reduced precipitation decreased plant species richness, while 50% increased precipitation did not significantly alter plant species richness. More importantly, we found that precipitation changes primarily regulated plant aboveground productivity by altering resource availability (water and inorganic nitrogen) and the size of dominant species. However, our experiment only focused on changes in the aboveground productivity of plant communities, with limited understanding of the responses of belowground plant productivity, soil animals, and soil microorganisms to precipitation changes. In future research, we will focus on the responses of the belowground productivity of plant communities and different trophic levels to precipitation changes.

## Figures and Tables

**Table 1:** Annual rainfall under different precipitation treatments from 2021 to 2024.

Year	CK	RP	IP
2021	227.9 mm	113.95 mm	341.85 mm
2022	313.2 mm	156.6 mm	469.8 mm
2023	264.1 mm	132.05 mm	396.15 mm
2024	332.4 mm	166.2 mm	498.6 mm

Note: CK, RP, and IP represent natural precipitation, 50% reduced precipitation, and 50% increased precipitation, respectively.

## Data Availability

Dataset available upon request from the authors.

## Supplementary Materials

The following supporting information can be downloaded at: https://www.mdpi.com/article/10.3390/biology14081010/s1, Table S1: The impact of precipitation changes on the relative abundance of grasses and forbs.
