# Supplementary material for: Effect of Precipitation Change on Desert Steppe Aboveground Productivity
Source: Biology (Basel). 2025 Aug 6;14(8):1010. doi: 10.3390/biology14081010 (PMC12383943; doi:10.3390/biology14081010)
Supplement: Supplementary file 1 [file biology-14-01010-s001.zip › biology-3739237-supplementary.pdf]

. Manuscript ID：biology-3739237

Manuscript Title: Effect of precipitation change on desert steppe aboveground productivity

Table S1 The impact of precipitation changes on the relative abundance of grasses and forbs. Note: CK, RP, and IP represent natural precipitation, 50% reduced precipitation, and 50% increased precipitation, respectively. Different uppercase letters indicate significant differences ($p < 0.05$).

	Grasses	Forbs
CK	0.21±0.08A	0.07±0.05AB
RP	0.08±0.08B	0.01±0.02B
IP	0.18±0.03AB	0.11±0.04A
